# Supplementary material for: On the Fate of Butyl Methoxydibenzoylmethane (Avobenzone) in Coral Tissue and Its Effect on Coral Metabolome
Source: Metabolites. 2023 Apr 7;13(4):533. doi: 10.3390/metabo13040533 (PMC10141135; doi:10.3390/metabo13040533)
Supplement: Supplementary file 1 [file metabolites-13-00533-s001.zip › metabolites-2283449-supplementary.pdf]

Supporting Information for:

On the fate of butyl methoxydibenzoylmethane (avobenzone) in coral tissue and its effect on coral metabolome

Fanny Clergeaud [1], Maeva Giraudo [1], Alice M. S. Rodrigues [1,2], Evane Thorel [1], Philippe Lebaron [1], Didier Stien [1,*]

Table of Content

Full experimental details 4
Table S1. ECOSAR v2.2 predicted toxicity of BM derivatives compared to BM (1) 5
Figure S1. Proposed structure of compound 2 6
Figure S2. ESI$^+$-MS spectrum showing ion [2+Na]$^+$ at *m/z* 619.3762, with expansion on the right 6
Figure S3. Collision-induced fragmentation spectrum of [2+Na]$^+$ at *m/z* 619.3762 6
Figure S4. Proposed structure of compound 3 6
Figure S5. ESI$^+$-MS spectrum showing ion [3+Na]$^+$ at *m/z* 595.3758 7
Figure S6. Collision-induced fragmentation spectrum of [3+Na]$^+$ at *m/z* 595.3758 7
Figure S7. Proposed structure of compound 4 7
Figure S8. ESI$^+$-MS spectrum showing ion [4+Na]$^+$ at *m/z* 645.3916 7
Figure S9. Collision-induced fragmentation spectrum of [4+Na]$^+$ at *m/z* 645.3916 7
Figure S10. Proposed structure of compound 5 8
Figure S11. ESI$^+$-MS spectrum showing ion [5+Na]$^+$ at *m/z* 621.3916 8
Figure S12. Collision-induced fragmentation spectrum of [5+Na]$^+$ at *m/z* 621.3916 8
Figure S13. Proposed structure of compound 6 8
Figure S14. ESI$^+$-MS spectrum showing ion [6+Na]$^+$ at *m/z* 623.4072 8
Figure S15. Collision-induced fragmentation spectrum of [6+Na]$^+$ at *m/z* 623.4072 9
Figure S16. Proposed structure of compound 7 9
Figure S17. ESI$^+$-MS spectrum showing ion [7+Na]$^+$ at *m/z* 573.3916 9
Figure S18. Collision-induced fragmentation spectrum of [7+Na]$^+$ at *m/z* 573.3916 9
Figure S19. Compared extracted ion chromatograms at *m/z* 573.3914 ([7+Na]$^+$) in an extract of BM-exposed coral (top) and synthetic analytical standard of compound 7 (bottom) 10
Figure S20. Compared collision-induced fragmentation spectra of [7+Na]$^+$ ion at *m/z* 573.3914 in an extract of BM-exposed coral (top) and synthetic analytical standard of compound 7 (bottom) 10
Figure S21. Proposed structure of compound 8 10
Figure S22. ESI$^+$-MS spectrum showing ion [8+Na]$^+$ at *m/z* 573.3916 11
Figure S23. Collision-induced fragmentation spectrum of [8+Na]$^+$ at *m/z* 573.3916 11
Figure S24. Proposed structure of compound 9 11
Figure S25. ESI$^+$-MS spectrum showing ion [9+Na]$^+$ at *m/z* 599.4071 11
Figure S26. Collision-induced fragmentation spectrum of [9+Na]$^+$ at *m/z* 599.4071 12
Figure S27. Proposed structure of compound 10 12
Figure S28. ESI$^+$-MS spectrum showing ion [10+Na]$^+$ at *m/z* 601.4229 12
Figure S29. Collision-induced fragmentation spectrum of [10+Na]$^+$ at *m/z* 601.4229 12
Figure S30. Proposed structure of compound 11 12

Figure S31. ESI$^+$-MS spectrum showing ion $[11+Na]^+$ at *m/z* 589.3864 13
Figure S32. Collision-induced fragmentation spectrum of $[11+Na]^+$ at *m/z* 589.3864 13
Figure S33. Proposed structure of compound 12 13
Figure S34. ESI$^+$-MS spectrum showing ion $[12+Na]^+$ at *m/z* 655.3033 13
Figure S35. Collision-induced fragmentation spectrum of $[12+Na]^+$ at *m/z* 655.3033 14
Figure S36. Proposed structure of compound 13 14
Figure S37. ESI$^+$-MS spectrum showing ion $[13+Na]^+$ at *m/z* 655.3034 14
Figure S38. Collision-induced fragmentation spectrum of $[13+Na]^+$ at *m/z* 655.3034 14
Figure S39. Collision-induced fragmentation spectrum of Frag. 1 at *m/z* 311.1643 15
Figure S40. Collision-induced fragmentation spectrum of Frag. 2 at *m/z* 323.1643 15
Figure S41. ESI$^+$-MS spectrum showing the undetermined ion at *m/z* 449.1370 corresponding to compound 14 15
Figure S42. Collision-induced fragmentation spectrum of ion at *m/z* 449.1370 15
Figure S43. ESI$^+$-MS spectrum showing ion $[15+H]^+$ at *m/z* 283.1694 16
Figure S44. Collision-induced fragmentation spectrum of $[15+H]^+$ at *m/z* 283.1694 16
Figure S45. ESI$^+$-MS spectrum showing ion $[16+Na]^+$ at *m/z* 559.3760 16
Figure S46. Collision-induced fragmentation spectrum of [16+Na]+ at m/z 559.3760 16
Figure S47. ESI$^+$-MS spectrum showing ion $[17+H]^+$ at *m/z* 279.1744 17
Figure S48. Collision-induced fragmentation spectrum of $[17+H]^+$ at *m/z* 279.1744 17
Table S2. Compared ESI$^+$-MS response of $[BM+H]^+$ and $[7+Na]^+$ ions, full data 17
Figure S49. Compared ESI$^+$-MS response of $[BM+H]^+$ and $[7+Na]^+$ ions in the range of concentration for which the response is linear 18
Table S3. Extracted ion chromatogram peak areas for compounds 1-11 in coral exposed to BM (data provided by Compound Discoverer), corrected peak areas to report on mass relative proportions, and relative corrected peak areas 19
Figure S50. ESI$^+$-MS spectrum showing ion $[18+H]^+$ at *m/z* 548.4674 20
Figure S51. Collision-induced fragmentation spectrum of $[18+H]^+$ at *m/z* 548.4674 20
Figure S52. ESI$^+$-MS spectrum showing ion $[19+H]^+$ at *m/z* 562.4830 20
Figure S53. Collision-induced fragmentation spectrum of $[19+H]^+$ at *m/z* 562.4830 20
Figure S54. ESI$^+$-MS spectrum showing ion $[20+H]^+$ at *m/z* 550.4832 21
Figure S55. Collision-induced fragmentation spectrum of $[20+H]^+$ at *m/z* 550.4832 21
Figure S56. ESI$^+$-MS spectrum showing ion $[21+H]^+$ at *m/z* 870.5731 21
Figure S57. Collision-induced fragmentation spectrum of $[21+H]^+$ at *m/z* 870.5731 21
Figure S58. ESI$^+$-MS spectrum showing ion $[22+H]^+$ at *m/z* 798.5731 22
Figure S59. Collision-induced fragmentation spectrum of $[22+H]^+$ at *m/z* 798.5731 22
Figure S60. Proposed structure of compound 23 22
Figure S61. ESI$^+$-MS spectrum showing ion $[23+Na]^+$ at *m/z* 789.4553 22
Figure S62. Collision-induced fragmentation spectrum of $[23+Na]^+$ at *m/z* 789.4553 23
Figure S63. Proposed structure of compound 24 23
Figure S64. ESI$^+$-MS spectrum showing ion $[24+Na]^+$ at *m/z* 791.4709 23
Figure S65. Collision-induced fragmentation spectrum of $[24+Na]^+$ at *m/z* 791.4709 23
Figure S66. Proposed structure of compound 25 24
Figure S67. ESI$^+$-MS spectrum showing ion $[25+Na]^+$ at *m/z* 817.4853 24
Figure S68. Collision-induced fragmentation spectrum of $[25+Na]^+$ at *m/z* 817.4866 24
Figure S69. Proposed structure of compound 26 24
Figure S70. ESI$^+$-MS spectrum showing ion $[26+Na]^+$ at *m/z* 769.4871 25
Figure S71. Collision-induced fragmentation spectrum of $[26+Na]^+$ at *m/z* 769.4871 25

Figure S72. Proposed structure of compound 27 25
Figure S73. ESI$^+$-MS spectrum showing ion [27+Na]$^+$ at *m/z* 771.5025.................................. 25
Figure S74. Collision-induced fragmentation spectrum of [27+Na]$^+$ at *m/z* 771.5025 26
Figure S75. ESI$^+$-MS spectrum showing ion [28+H]$^+$ at *m/z* 613.3521.................................... 26
Figure S76. Collision-induced fragmentation spectrum of [28+H]$^+$ at *m/z* 613.3521 26
Figure S77. ESI$^+$-MS spectrum showing ion [29+H]$^+$ at *m/z* 613.3525.................................... 26
Figure S78. Collision-induced fragmentation spectrum of [29+H]$^+$ at *m/z* 613.3521 27
Figure S79. ESI$^+$-MS spectrum showing ion [30+NH_4]$^+$ at *m/z* 1070.6836.............................. 27
Figure S80. Collision-induced fragmentation spectrum of [30+NH_4]$^+$ at *m/z* 1070.6836 27

Full experimental details

Pocillopora damicornis. Samples of the coral *P. damicornis* were collected in Oman and were acclimated for more than one year in tanks at the Banyuls Oceanological Observatory. Corals were maintained in artificial seawater (ASW) prepared with reverse osmosis purified water and Reef Salt SeaChem salts. Salinity was adjusted to 36 g/L, pH = 8, and the temperature was set at 24 °C. All experiments were conducted with the same ASW.

Preparation of Coral for Exposition to Solar Filters. Coral pieces (1−1.5 cm) were cut from branch tips from the same mother colony. Each piece was attached to a fishing line, and the coral pieces were then suspended each on one ∼5−6 cm plastic grid, making sure the lines had the appropriate length so that the coral pieces will stand in the middle of exposure beakers during the assay. Each suspended coral nubbin (five replicates) was clamped to grid above the aquarium of origin, thereby immerging the pieces. The suspended pieces were acclimatized for approximately 1 month before the assay to allow for full healing.

Exposure Setup. Beakers (250 mL) were filled with ASW (200 mL). BM (≥98.0%, Sigma-Aldrich) was dissolved in DMSO at concentrations of 400, 120, 20, and 2 µg/mL. Each of these solutions (500 µL) was added into separate beakers, resulting in final concentrations of 1000, 300, 50, and 5 µg/L. DMSO (500 µL) was used as a negative control, and the DMSO concentration was 0.25% v/v in all beakers. The suspended coral pieces were dipped into the beakers, with the grid positioning the pieces halfway into the water. Gentle bubbling in the beakers was maintained using aquarium air pumps equipped with a Teflon tubing with Pasteur pipettes tips. Homogeneous lighting was set to 250 µmol/m^2/s, with a cycle of 10 h of day and 14 h of night. The corals were transferred every 24 h to a new beaker prepared in the same way. The total exposure lasted for 7 days. Pictures were taken every 24 h to monitor coral polyps. Coral exposure at 1000 µg/L BM was repeated in a separate experiment to confirm the result.

Extraction. After 7 days, the coral pieces were collected and the fishing lines were removed with a scalpel. Each coral piece was placed into a 20 mL test tube, covered with MeOH/ acetonitrile 1/1, and sonicated for 20 min. Once the polyps were dissolved, the solvent was evaporated and the dry samples were kept at −80 °C until analysis. All solvents used for extraction and profiling were LC-MS gradient grade (Biosolve, Dieuze, France).

UHPLC-HRMS Profiling. High-resolution MS/MS analyses were conducted with a Thermo UHPLC-HRMS system. Analyses were performed in the electrospray positive ionization mode in the range of 133.4−2000 Da in centroid mode. The mass detector was an Orbitrap MS/MS FT Q-Exactive focus mass spectrometer. The analyses were conducted in FullMS data dependent MS^2 mode. In FullMS, the resolution was set to 70000, and the AGC target was 3 × 10^6. InMS^2, the resolution was 17500, AGC target 10^5, isolation window 0.4 Da, and stepped normalized collision energy 15/30/45 was used, with 6 s dynamic exclusion. The lock mass option was set for ion at m/z 144.98215, corresponding to $Cu(CH_3CN)^{2+}$. The UHPLC column was a Phenomenex Luna Omega polar C-18 150 mm × 2.1 mm, 1.6 µm. The column temperature was set to 42 °C, and the flow rate was 0.5 mL·min^{-1}. The solvent system was a mixture of water (solution A) with increasing proportions of acetonitrile (solution B), both solvents modified with 0.1% formic acid. The gradient was as follows: 2%

B 3 min before injection, then from 1 to 13 min, a shark fin gradient increase of B up to 100% (curve 2), followed by 100% B for 5 min. The flow was diverted (not injected into the mass spectrometer) before injection, up to 1 min after injection. The coral extracts were dissolved in MeOH (1 mg/mL) by sonication, and 2 µL was injected onto the column. Standard of sythetic palmitic acid dihydroBM ester (**7**) in acetonitrile (0.1 µg/mL, 1 µL injected) was analyzed with the same method.

Metabolomic Analyses. Profiles were analyzed with Compound Discoverer (CD) 2.1 (ThermoFisher, Villebon, France). Coral exposed to DMSO was used as the reference metabolome, and blank injections were used to remove irrelevant ion peaks (ion peak is considered only if the signal is at least 5 times more intense than in the blank). An untargeted metabolomic workflow was used. This workflow finds and identifies the differences between samples and performs retention time alignment, unknown compound detection, and compound grouping across all samples. The workflow predicts elemental compositions for all compounds, fills gaps across all samples, hides chemical background (using Blank samples), identifies compounds using mzCloud (ddMS^2) and ChemSpider (formula or exact mass), performs similarity searches for all compounds with ddMS2 data using mzCloud, and calculates a differential analysis (t-test or ANOVA) and determines p values, adjusted p values, ratios, and fold change. The retention time window was set to 2−17 min. The maximum shift for alignment was 0.1 min, the maximum mass tolerance was 2 ppm and the minimum peak intensity was 2×10^6. Statistical analyses (Principal Component Analysis [PCA], volcano plots) were performed with CD using centered and scaled data.

Predicted toxicity of BM derivatives compared to BM. The prediction of BM derivatives toxicity in aquatic organisms was carried out using an ecological structure-activity relationships (ECOSAR) predictive model. This model estimates acute and chronic toxicity concentrations for aquatic organisms by analyzing chemical structure similarity, using data reported to USEPA. To accomplish this task, ECOSAR V 2.2 was utilized in the study, which is distributed by the EPA (https://www.epa.gov/tsca-screening-tools/ecological-structure-activity-relationships-ecosar-predictive-model). For acute toxicity, LC50 values on green algae (EC_{50} at 96 h), daphnids (LC_{50} at 48 h), and fish (LC_{50} at 96 h) were considered. ECOSAR also generated predicted chronic toxicity information (Chronic Value, ChV) using the geometric mean of the predicted lowest observed effect concentration (LOEC) and the predicted no observed effect concentration (NOEC). Results for compounds **1**, **4**, **7**, **11** and **12** and reported in Table S1.

Table S1. ECOSAR v2.2 predicted toxicity of BM derivatives compared to BM (**1**)

	**Acute (mg/L)**			**Chronic (mg/L)**		
**Cmpd.**	**Fish**	**Daphnid**	**Green Algae**	**Fish**	**Daphnid**	**Green Algae**
**1**	0.64	0.71	0.26	0.036	0.034	0.16
**4**	5.2×10^{-06}	5.1×10^{-06}	2.7×10^{-07}	1.4×10^{-06}	5.6×10^{-06}	2.5×10^{-07}
**7**	4.6×10^{-05}	5.1×10^{-05}	3.7×10^{-06}	1.0×10^{-05}	4.2×10^{-05}	2.9×10^{-06}
**11**	4.0×10^{-04}	5.0×10^{-04}	4.9×10^{-05}	7.3×10^{-05}	3.1×10^{-04}	3.3×10^{-05}
**12**	0.0026	0.0051	0.0039	8.6×10^{-05}	2.7×10^{-04}	0.0055

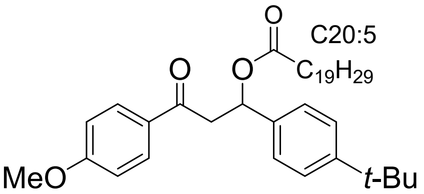

Figure S1. Proposed structure of compound **2**

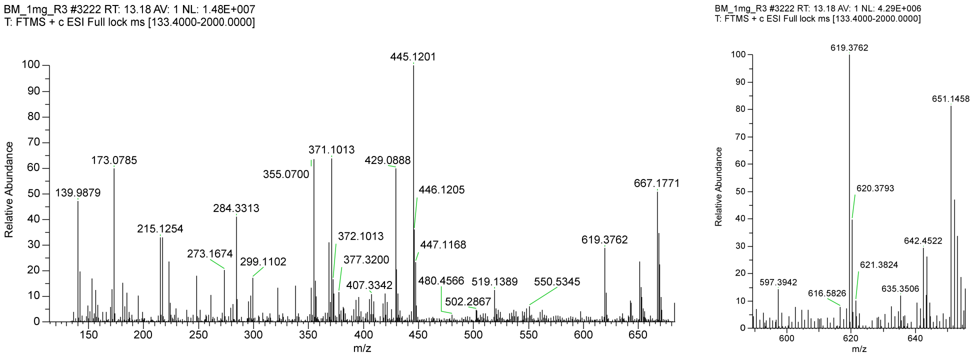

Figure S2. ESI$^+$-MS spectrum showing ion [**2**+Na]$^+$ at *m/z* 619.3762, with expansion on the right

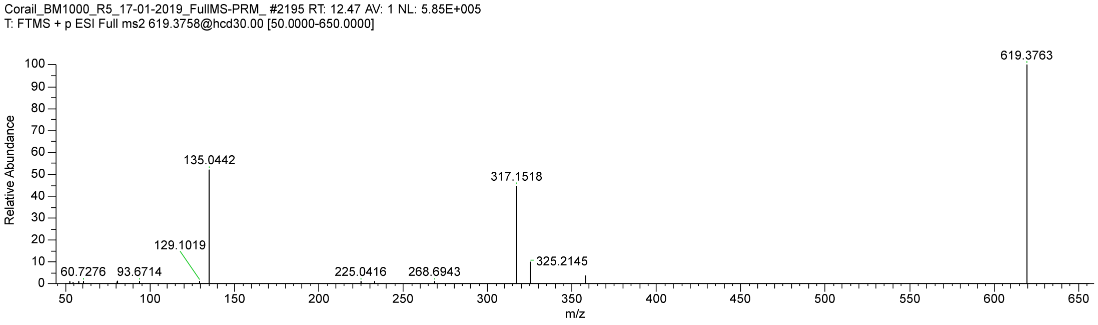

Figure S3. Collision-induced fragmentation spectrum of [**2**+Na]$^+$ at *m/z* 619.3762

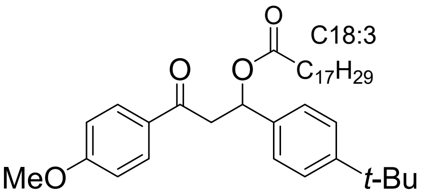

Figure S4. Proposed structure of compound **3**

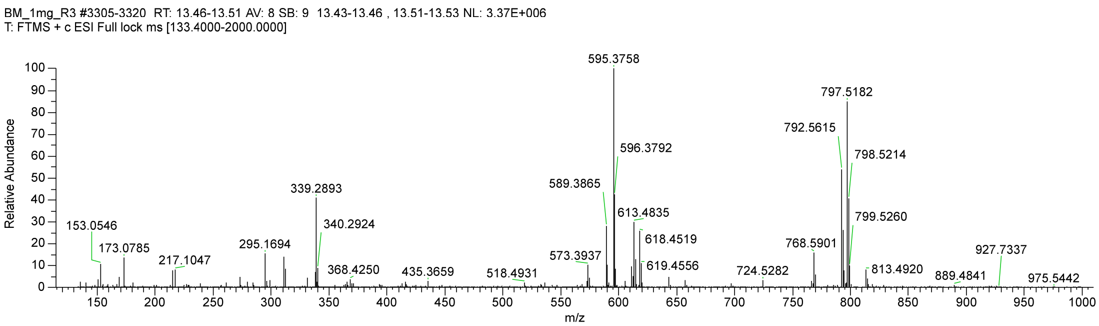

Figure S5. ESI$^+$-MS spectrum showing ion [**3**+Na]$^+$ at *m/z* 595.3758

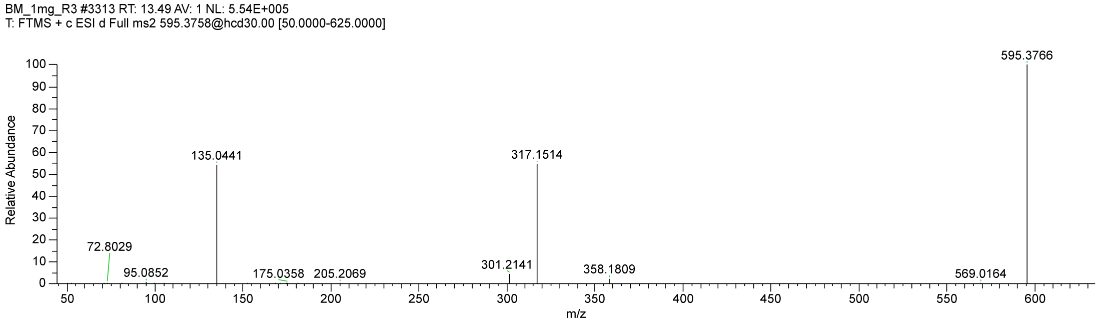

Figure S6. Collision-induced fragmentation spectrum of [**3**+Na]$^+$ at *m/z* 595.3758

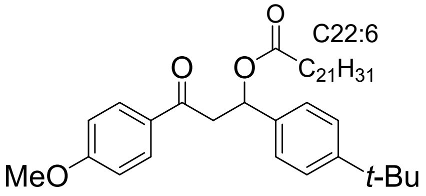

Figure S7. Proposed structure of compound **4**

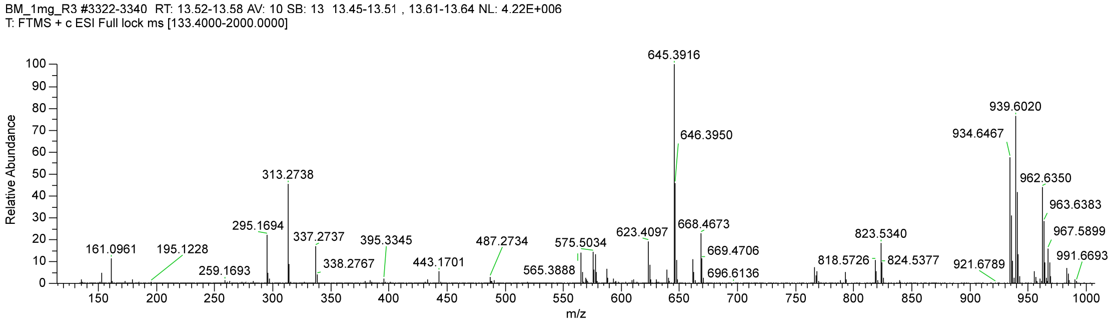

Figure S8. ESI$^+$-MS spectrum showing ion [**4**+Na]$^+$ at *m/z* 645.3916

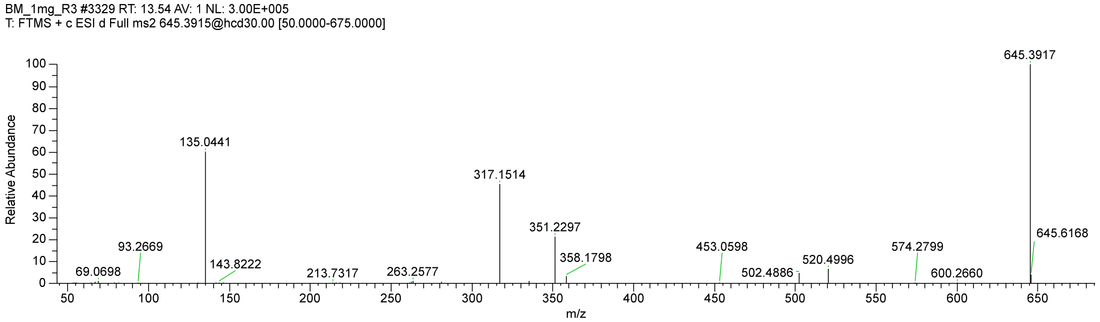

Figure S9. Collision-induced fragmentation spectrum of [**4**+Na]$^+$ at *m/z* 645.3916

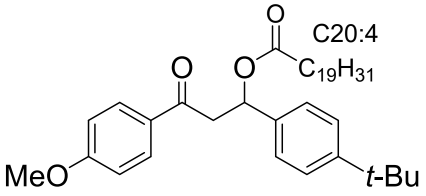

Figure S10. Proposed structure of compound **5**

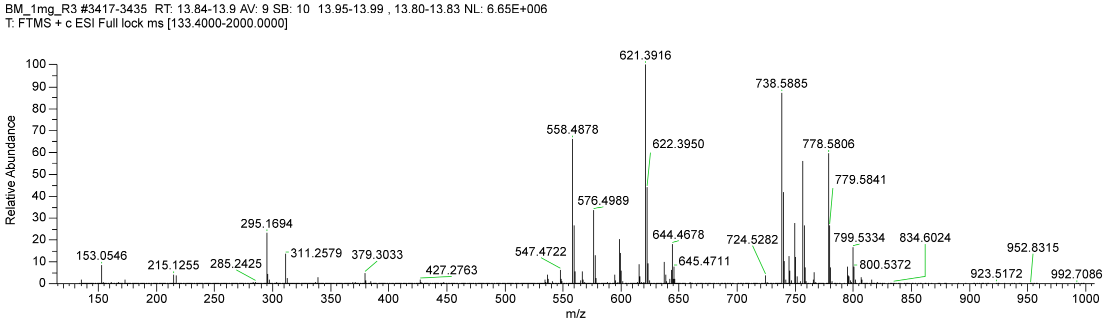

Figure S11. ESI$^+$-MS spectrum showing ion [**5**+Na]$^+$ at *m/z* 621.3916

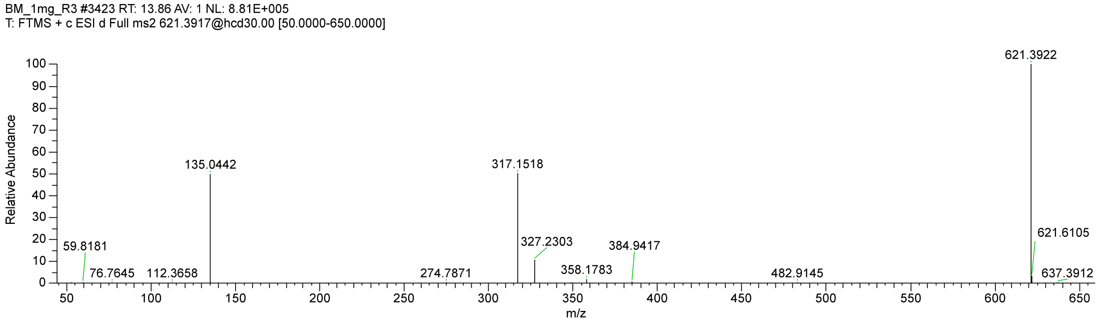

Figure S12. Collision-induced fragmentation spectrum of [**5**+Na]$^+$ at *m/z* 621.3916

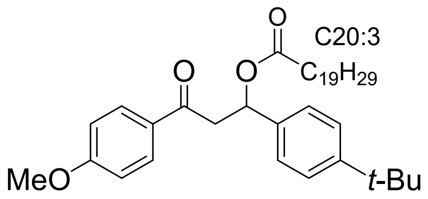

Figure S13. Proposed structure of compound **6**

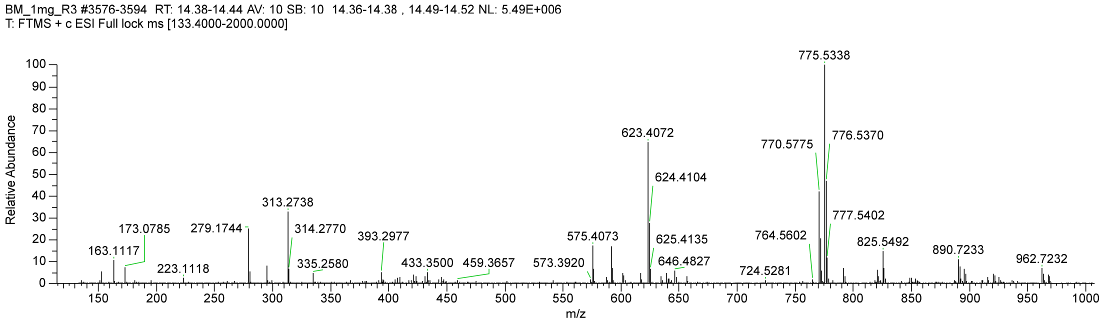

Figure S14. ESI$^+$-MS spectrum showing ion [**6**+Na]$^+$ at *m/z* 623.4072

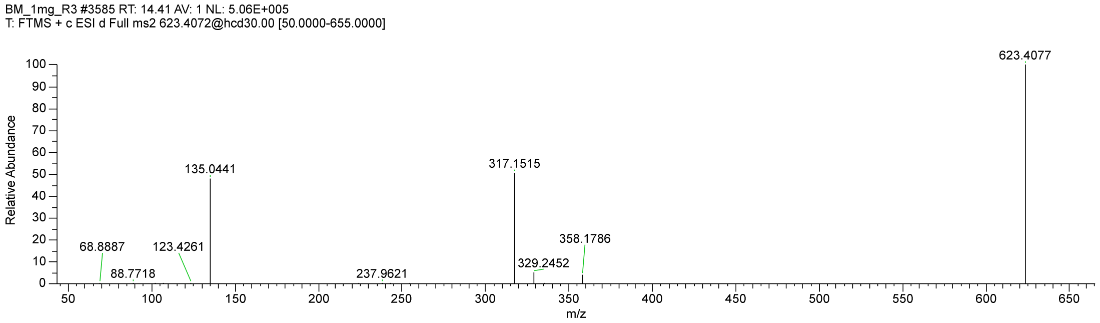

Figure S15. Collision-induced fragmentation spectrum of [**6**+Na]$^+$ at *m/z* 623.4072

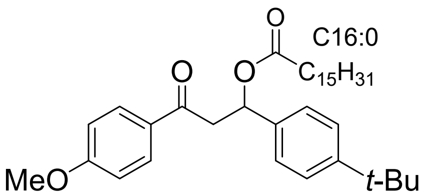

Figure S16. Proposed structure of compound **7**

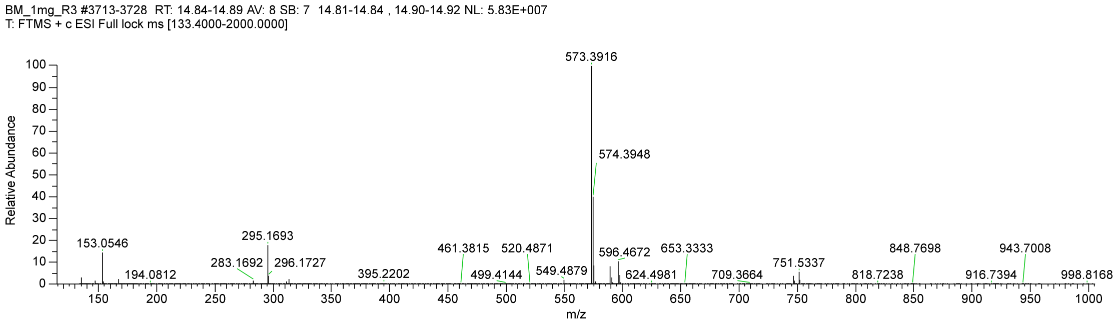

Figure S17. ESI$^+$-MS spectrum showing ion [**7**+Na]$^+$ at *m/z* 573.3916

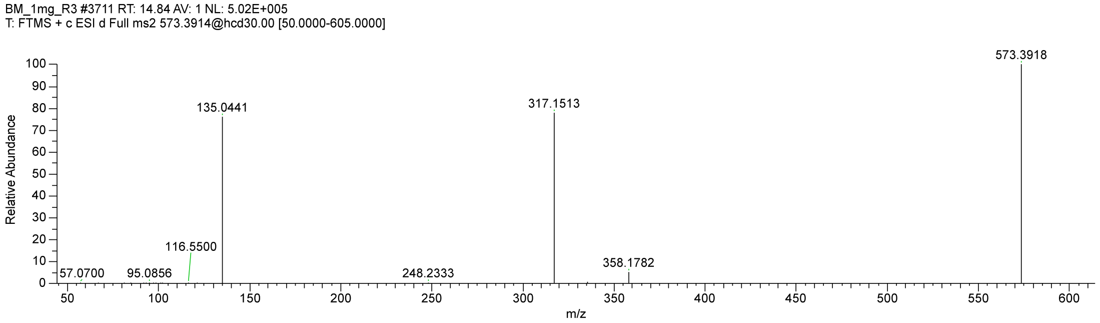

Figure S18. Collision-induced fragmentation spectrum of [**7**+Na]$^+$ at *m/z* 573.3916

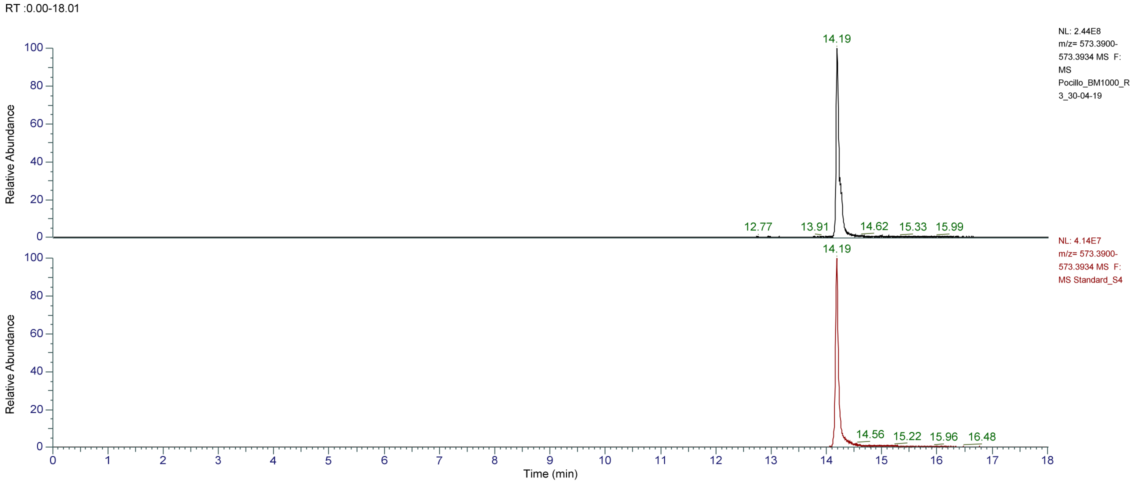

Figure S19. Compared extracted ion chromatograms at *m/z* 573.3914 ([**7**+Na]+) in an extract of BM-exposed coral (top) and synthetic analytical standard of compound **7** (bottom)

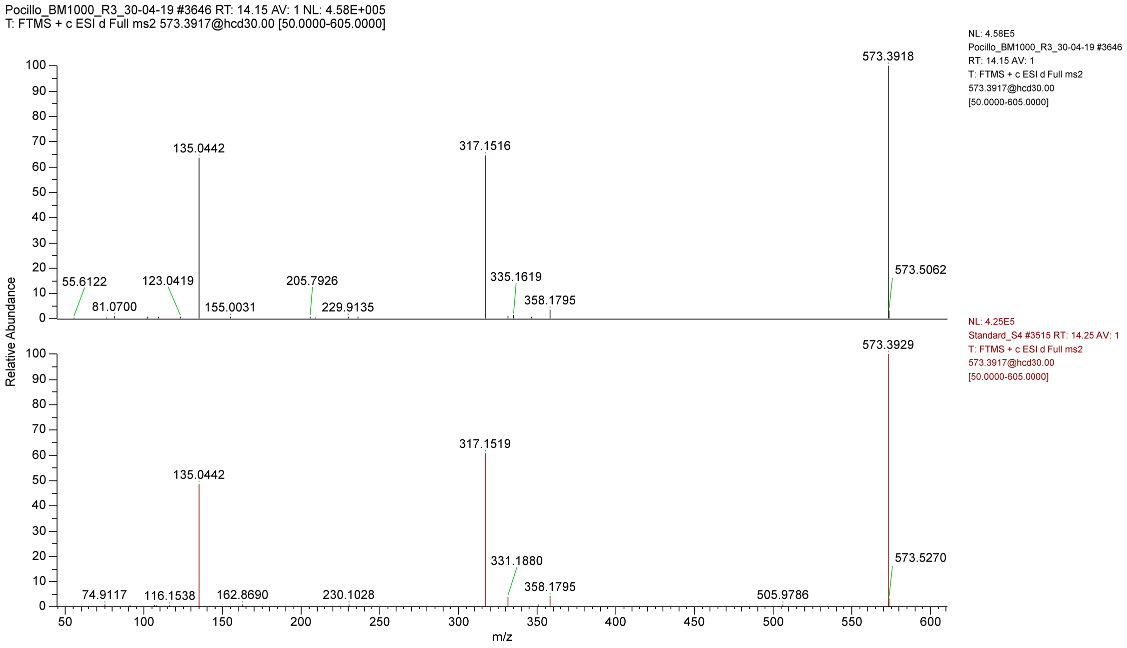

Figure S20. Compared collision-induced fragmentation spectra of [**7**+Na]+ ion at *m/z* 573.3914 in an extract of BM-exposed coral (top) and synthetic analytical standard of compound **7** (bottom)

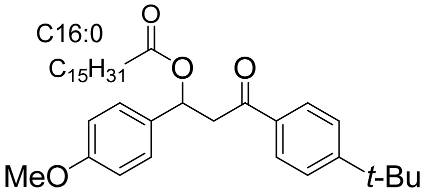

Figure S21. Proposed structure of compound **8**

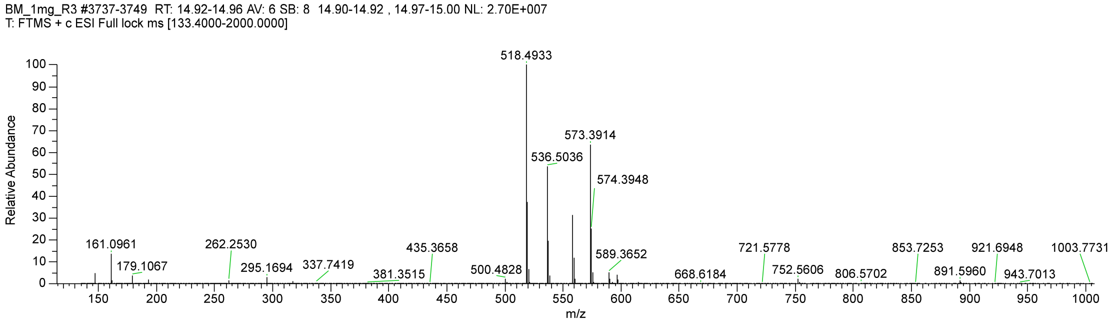

Figure S22. ESI$^+$-MS spectrum showing ion [**8**+Na]$^+$ at *m/z* 573.3916

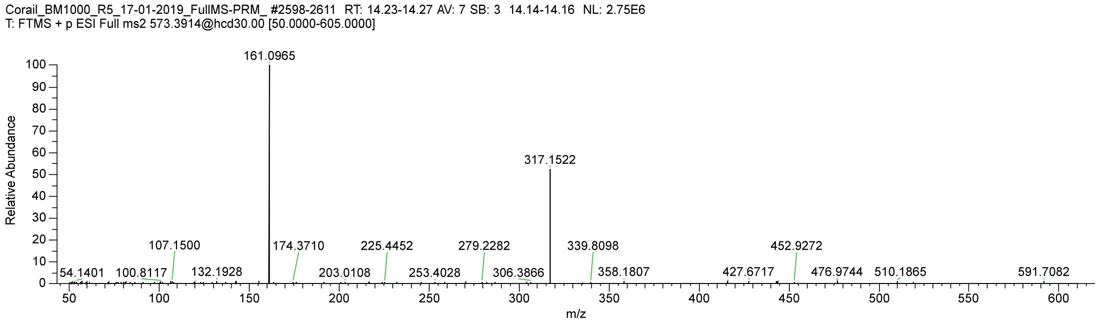

Figure S23. Collision-induced fragmentation spectrum of [**8**+Na]$^+$ at *m/z* 573.3916

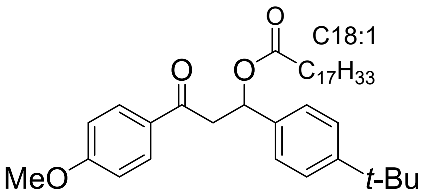

Figure S24. Proposed structure of compound **9**

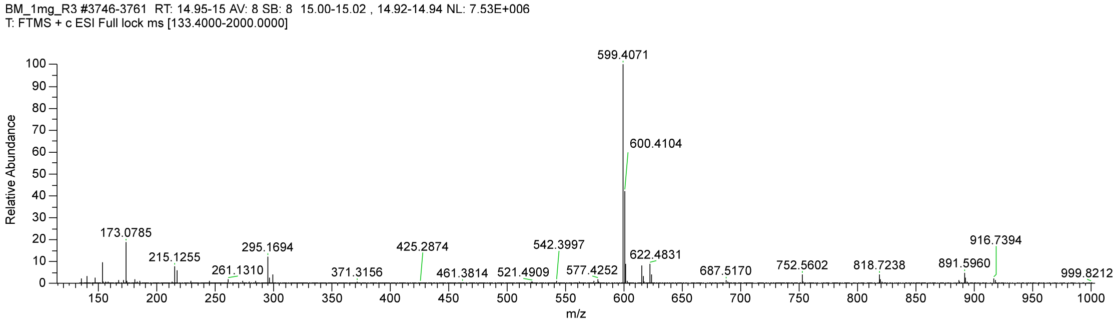

Figure S25. ESI$^+$-MS spectrum showing ion [**9**+Na]$^+$ at *m/z* 599.4071

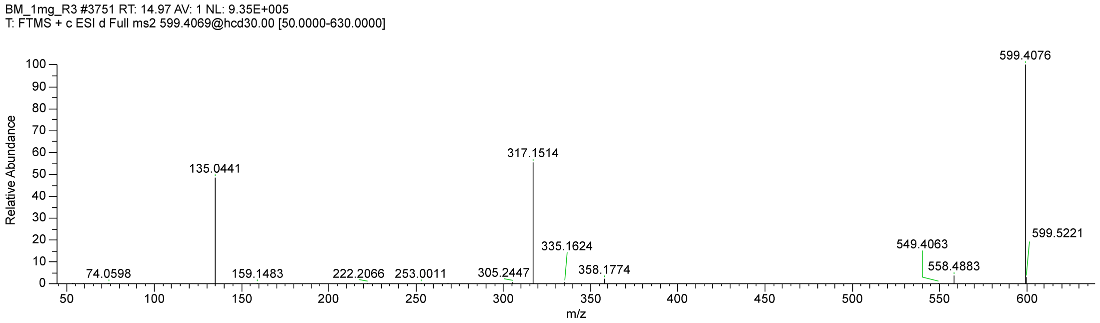

Figure S26. Collision-induced fragmentation spectrum of [**9**+Na]$^+$ at *m/z* 599.4071

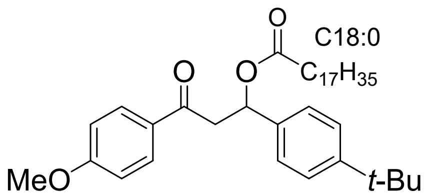

Figure S27. Proposed structure of compound **10**

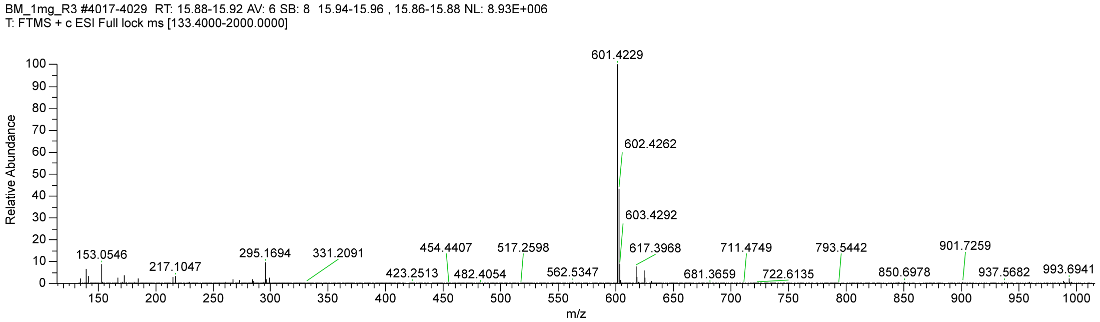

Figure S28. ESI$^+$-MS spectrum showing ion [**10**+Na]$^+$ at *m/z* 601.4229

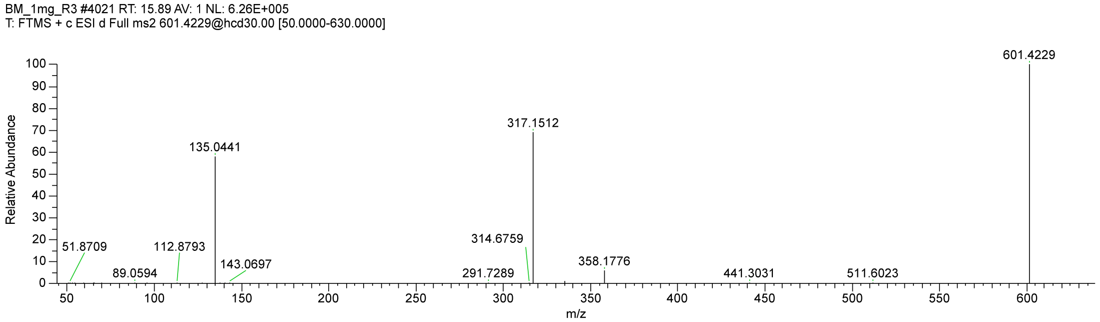

Figure S29. Collision-induced fragmentation spectrum of [**10**+Na]$^+$ at *m/z* 601.4229

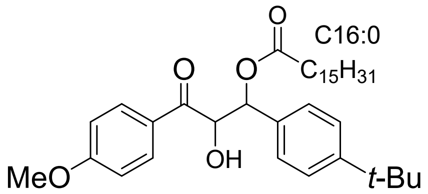

Figure S30. Proposed structure of compound **11**

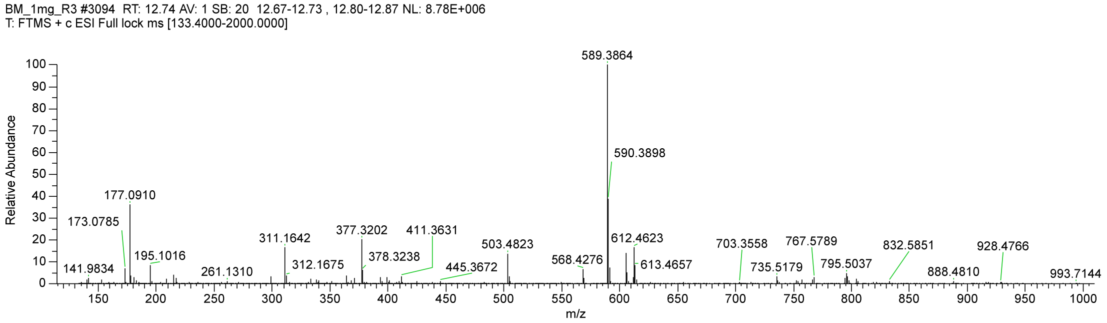

Figure S31. ESI$^+$-MS spectrum showing ion [**11**+Na]$^+$ at *m/z* 589.3864

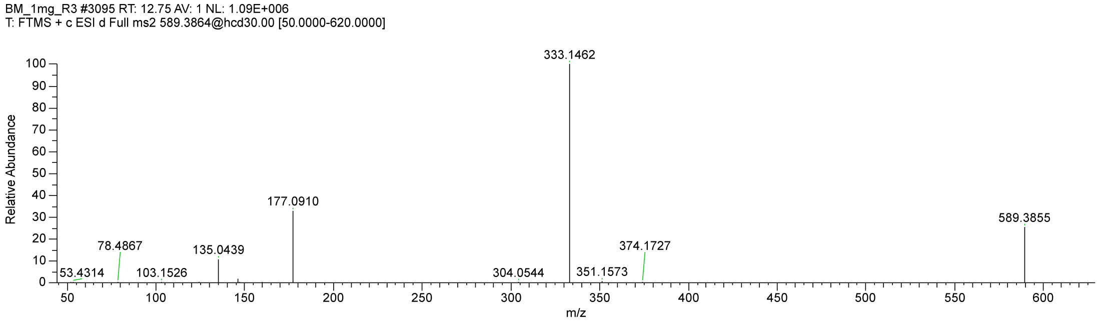

Figure S32. Collision-induced fragmentation spectrum of [**11**+Na]$^+$ at *m/z* 589.3864

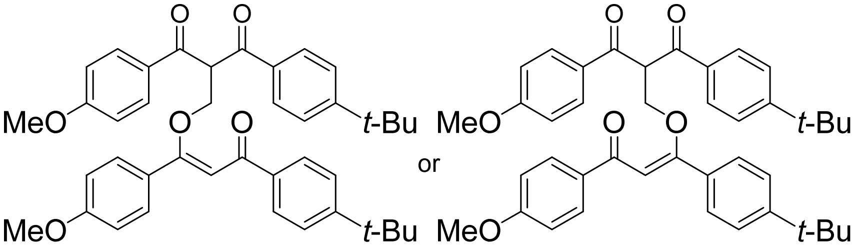

Figure S33. Proposed structure of compound **12**

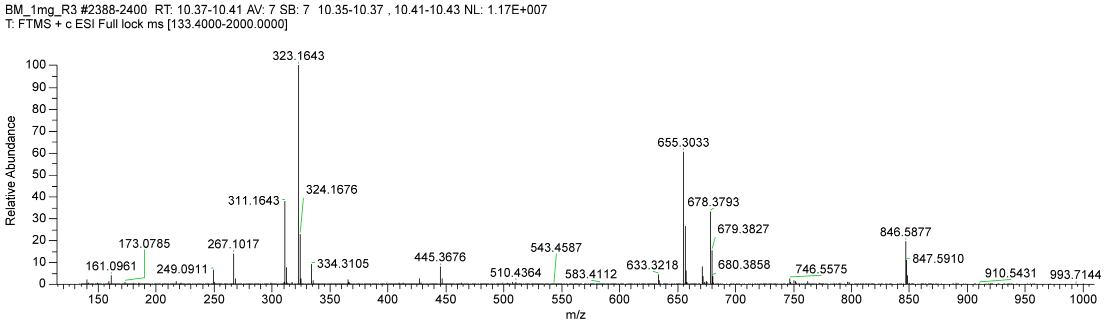

Figure S34. ESI$^+$-MS spectrum showing ion [**12**+Na]$^+$ at *m/z* 655.3033

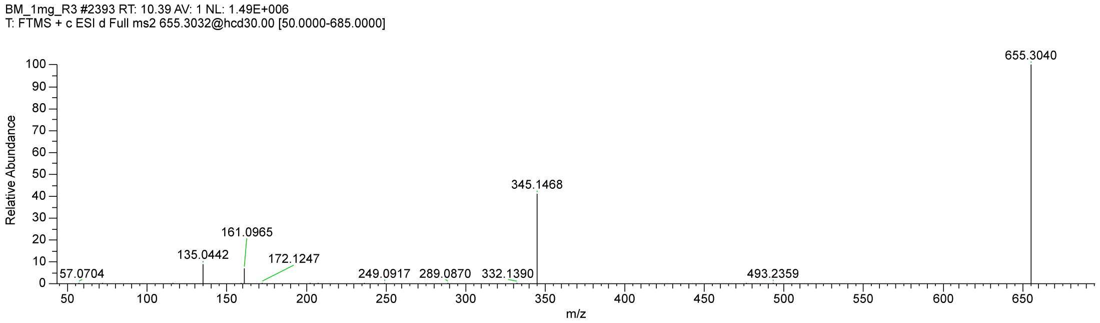

Figure S35. Collision-induced fragmentation spectrum of [**12**+Na]$^+$ at *m/z* 655.3033

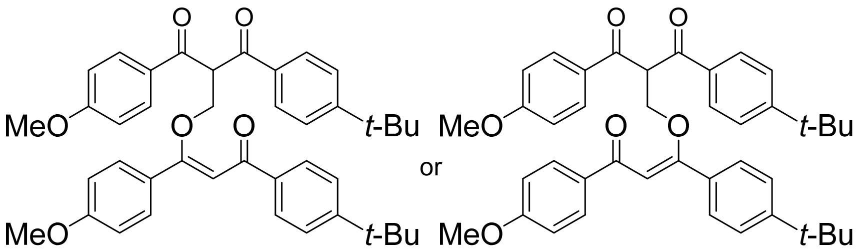

Figure S36. Proposed structure of compound **13**

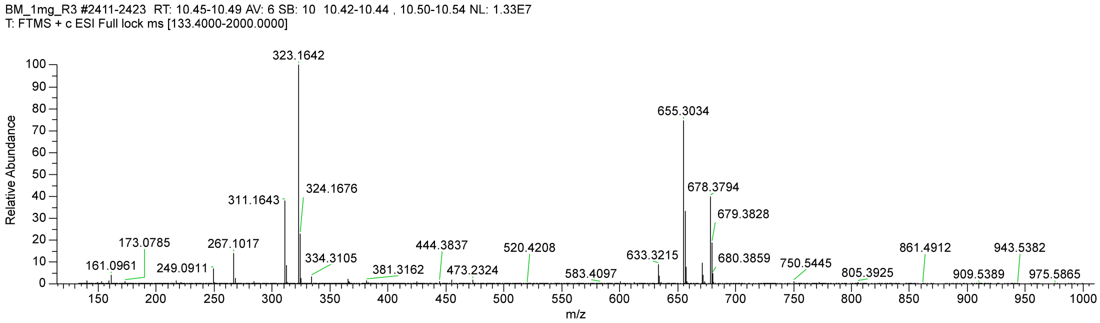

Figure S37. ESI$^+$-MS spectrum showing ion [**13**+Na]$^+$ at *m/z* 655.3034

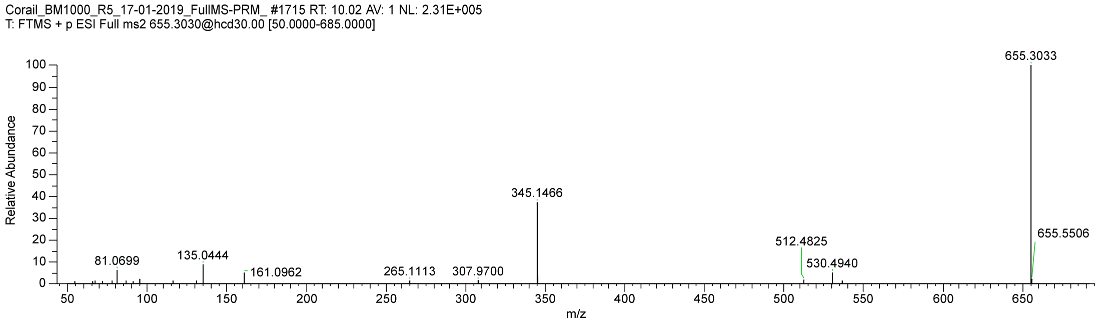

Figure S38. Collision-induced fragmentation spectrum of [**13**+Na]$^+$ at *m/z* 655.3034

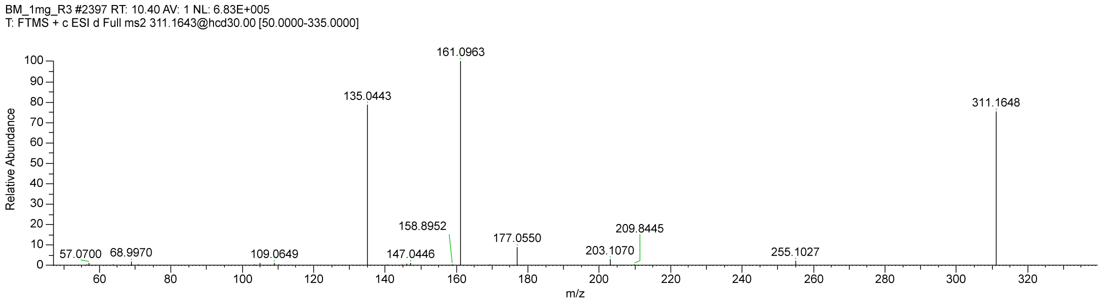

Figure S39. Collision-induced fragmentation spectrum of Frag. 1 at *m/z* 311.1643

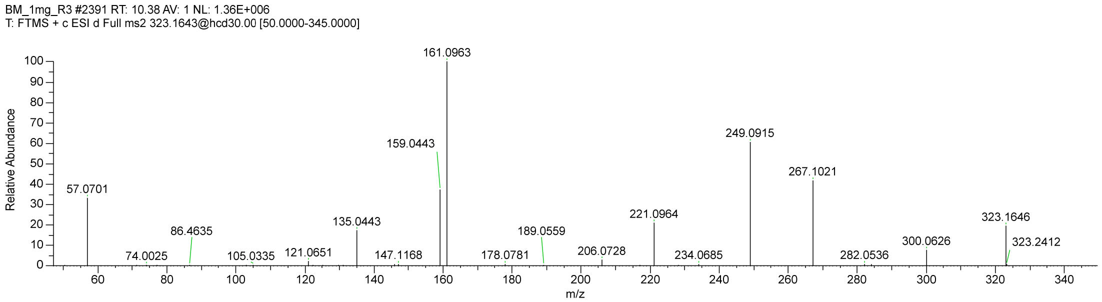

Figure S40. Collision-induced fragmentation spectrum of Frag. 2 at *m/z* 323.1643

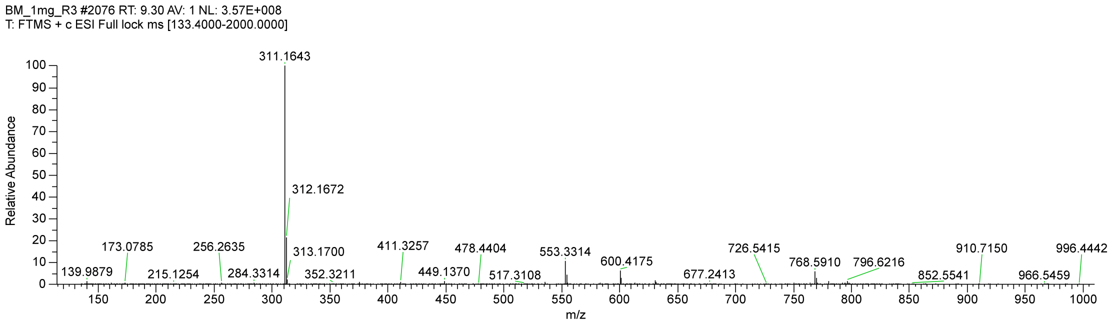

Figure S41. ESI$^+$-MS spectrum showing the undetermined ion at *m/z* 449.1370 corresponding to compound **14**

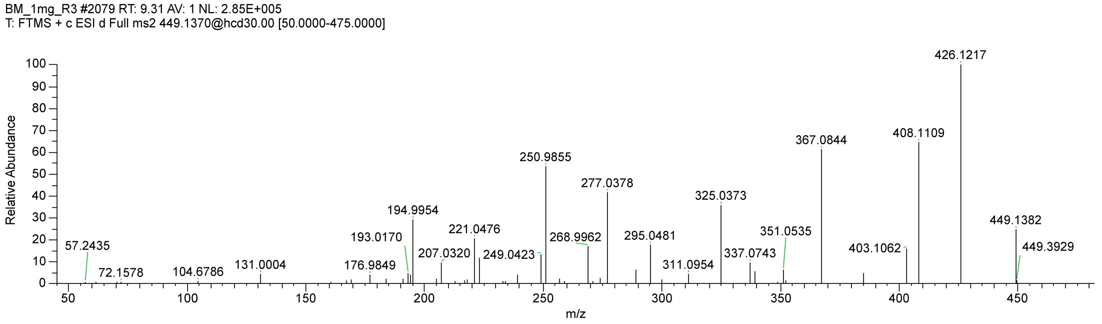

Figure S42. Collision-induced fragmentation spectrum of ion at *m/z* 449.1370

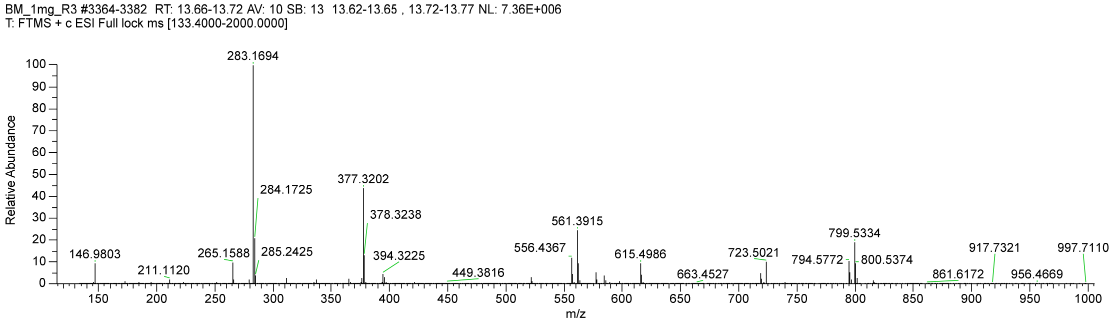

Figure S43. ESI$^+$-MS spectrum showing ion [**15**+H]$^+$ at *m/z* 283.1694

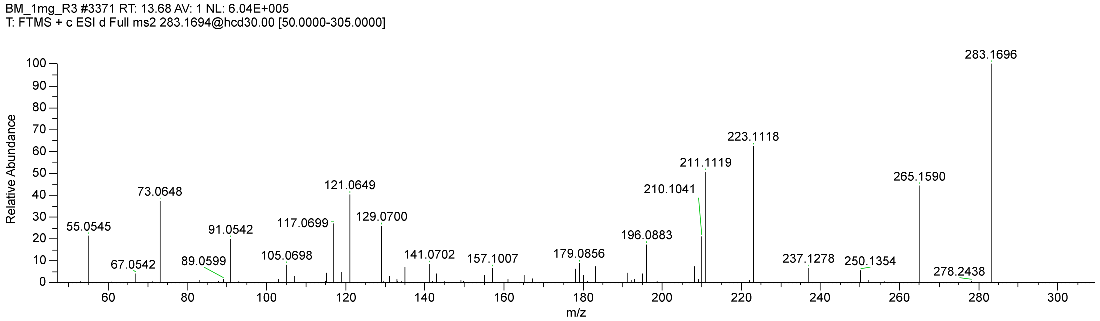

Figure S44. Collision-induced fragmentation spectrum of [**15**+H]$^+$ at *m/z* 283.1694

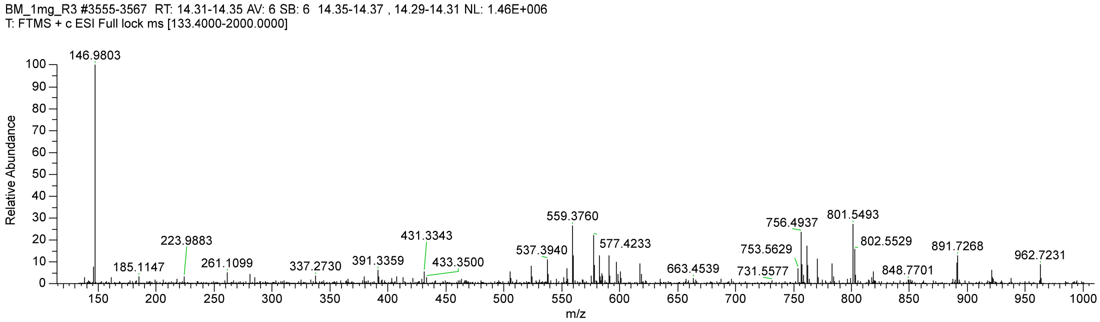

Figure S45. ESI$^+$-MS spectrum showing ion [**16**+Na]$^+$ at *m/z* 559.3760

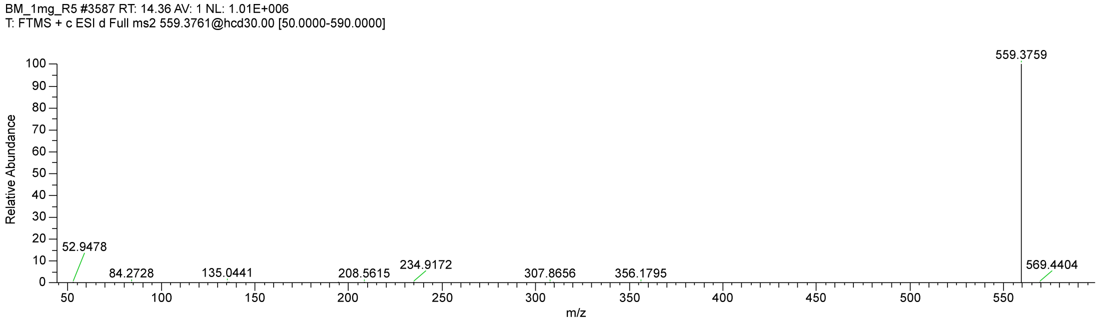

Figure S46. Collision-induced fragmentation spectrum of [**16**+Na]+ at m/z 559.3760

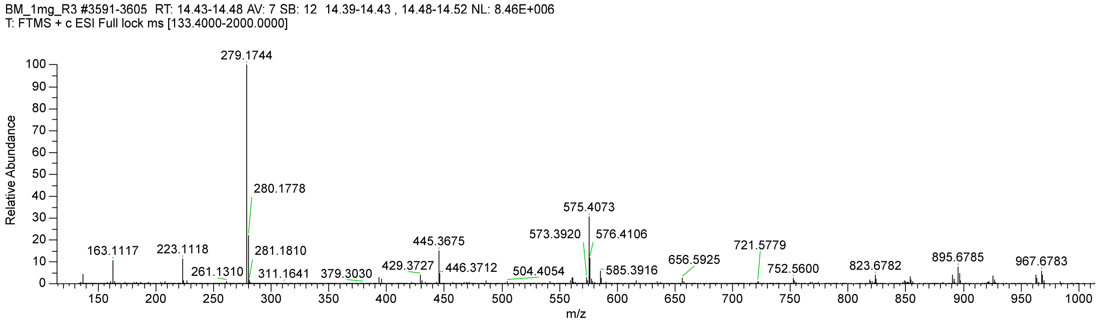

Figure S47. ESI$^+$-MS spectrum showing ion [**17**+H]$^+$ at *m/z* 279.1744

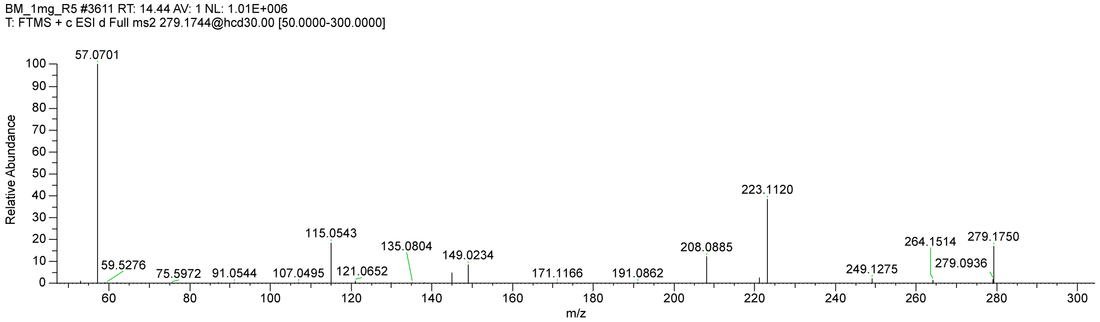

Figure S48. Collision-induced fragmentation spectrum of [**17**+H]$^+$ at *m/z* 279.1744

Table S2. Compared ESI$^+$-MS response of [BM+H]$^+$ and [**7**+Na]$^+$ ions, full data

	**BM**	**C16:0-dihydroBM**
Ion	**[M+H]$^+$, major tautomer**	**[M+Na]$^+$**
m/z	311.1642	573.3914
Initial mass (mg)	2.8	2.1
Relative mass	1	0.75
Concentration in S2 (µg/mL)	14.7	11.1
Area in S2	6.52E+09	4.96E+09
Relative response in MS at ~ 12 µg/mL	1	**1.02**
Concentration in S3 (µg/mL)	1.47	1.11
Area in S3	5.62E+08	1.22E+09
Relative response in MS at ~ 1.2 µg/mL	1	**2.90**
Concentration in S4 (µg/mL)	0.147	0.111
Area in S4	5.36E+07	1.74E+08
Relative response in MS at ~ 0.12 µg/mL	1	**4.33**
Concentration in S5 (µg/mL)	0.0147	0.0111
Area in S4	7.49E+06	1.79E+07
Relative response in MS at ~ 0.012 µg/mL	1	**3.18**

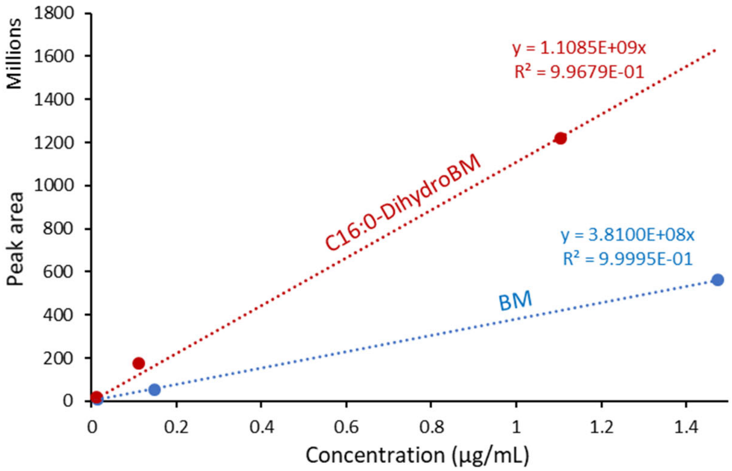

Figure S49. Compared ESI$^+$-MS response of $[BM+H]^+$ and $[\mathbf{7}+Na]^+$ ions in the range of concentration for which the response is linear

Table S3. Extracted ion chromatogram peak areas for compounds **1**-**11** in coral exposed to BM (data provided by Compound Discoverer), corrected peak areas to report on mass relative proportions, and relative corrected peak areas

	[BM] (μg/L)	Cmpd. #											Total 2-11
		**1 (BM)**	**2**	**3**	**4**	**5**	**6**	**7**	**8**	**9**	**10**	**11**	
Peak area in coral exposed to BM	**5**	26138	2610	3311	2736	2217	2778	52070	20102	2508	4270	5460	**98063**
	**50**	14927	5450	3825	8771	20132	6028	429584	13318	4464	32055	11363	**534990**
	**300**	60696	98042	81898	182842	140004	63314	4423157	50338	85529	450488	338275	**5913886**
	**1000**	434362	224511	440193	584738	592647	334674	16668663	342890	415686	1154072	1035388	**21793462**
Corrected peak area in coral exposed to BM (For compounds **2**-**11**, Peak area/3)	**5**	26138	870	1104	912	739	926	17357	6701	836	1423	1820	**32688**
	**50**	14927	1817	1275	2924	6711	2009	143195	4439	1488	10685	3788	**178330**
	**300**	60696	32681	27299	60947	46668	21105	1474386	16779	28510	150163	112758	**1971295**
	**1000**	434362	74837	146731	194913	197549	111558	5556221	114297	138562	384691	345129	**7264487**
Relative corrected peak area (in relation to BM peak area) in coral exposed to BM	**5**		0.03	0.04	0.03	0.03	0.04	0.66	0.26	0.03	0.05	0.07	**1.3**
	**50**		0.12	0.09	0.20	0.45	0.13	9.59	0.30	0.10	0.72	0.25	**12**
	**300**		0.54	0.45	1.00	0.77	0.35	24.29	0.28	0.47	2.47	1.86	**32**
	**1000**		0.17	0.34	0.45	0.45	0.26	12.79	0.26	0.32	0.89	0.79	**17**

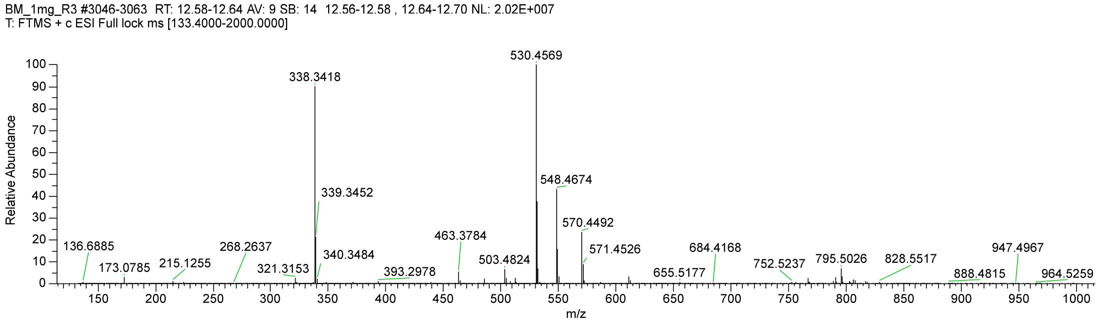

Figure S50. ESI$^+$-MS spectrum showing ion [**18**+H]$^+$ at *m/z* 548.4674

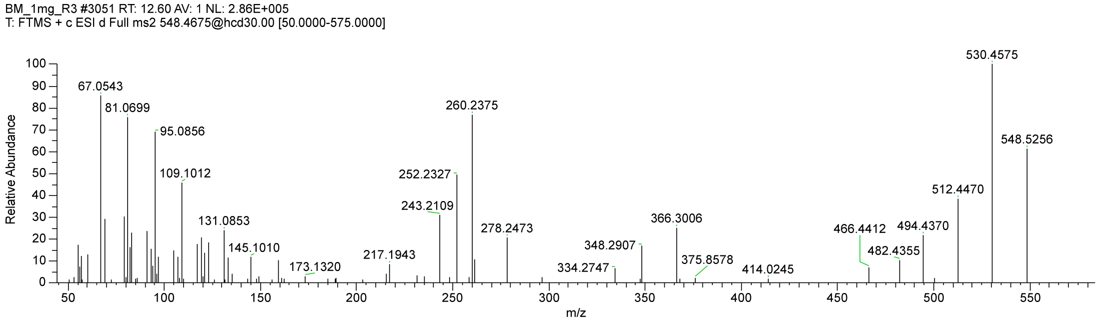

Figure S51. Collision-induced fragmentation spectrum of [**18**+H]$^+$ at *m/z* 548.4674

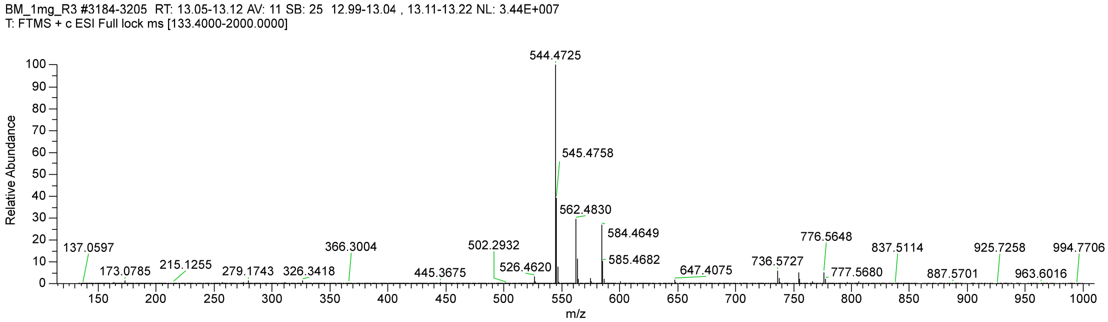

Figure S52. ESI$^+$-MS spectrum showing ion [**19**+H]$^+$ at *m/z* 562.4830

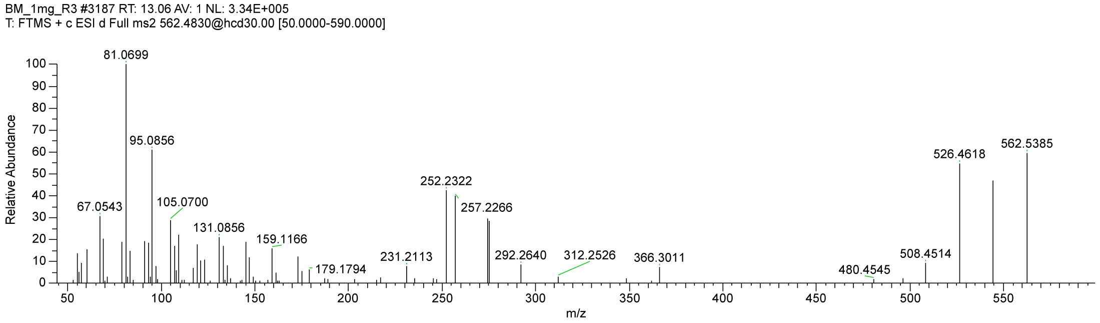

Figure S53. Collision-induced fragmentation spectrum of [**19**+H]$^+$ at *m/z* 562.4830

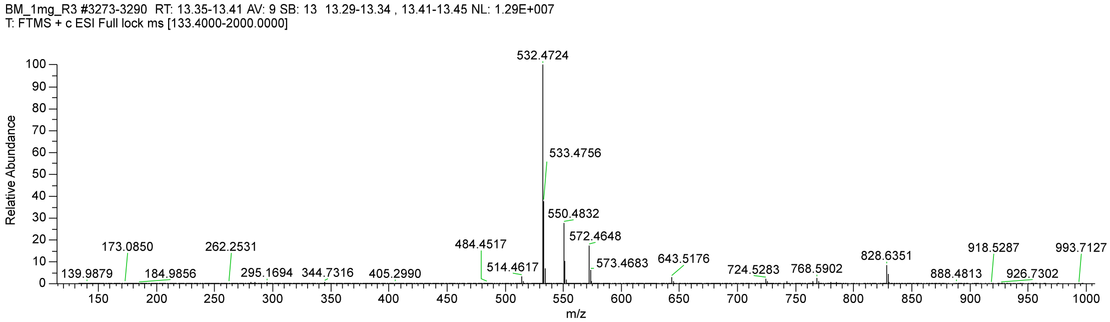

Figure S54. ESI$^+$-MS spectrum showing ion [**20**+H]$^+$ at *m/z* 550.4832

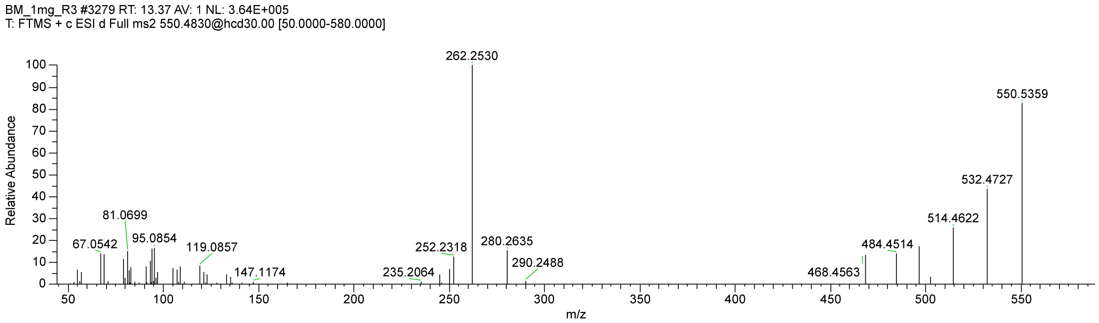

Figure S55. Collision-induced fragmentation spectrum of [**20**+H]$^+$ at *m/z* 550.4832

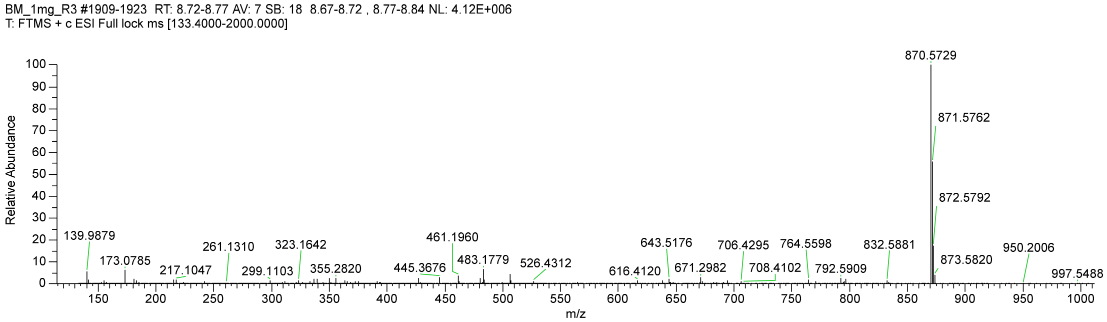

Figure S56. ESI$^+$-MS spectrum showing ion [**21**+H]$^+$ at *m/z* 870.5731

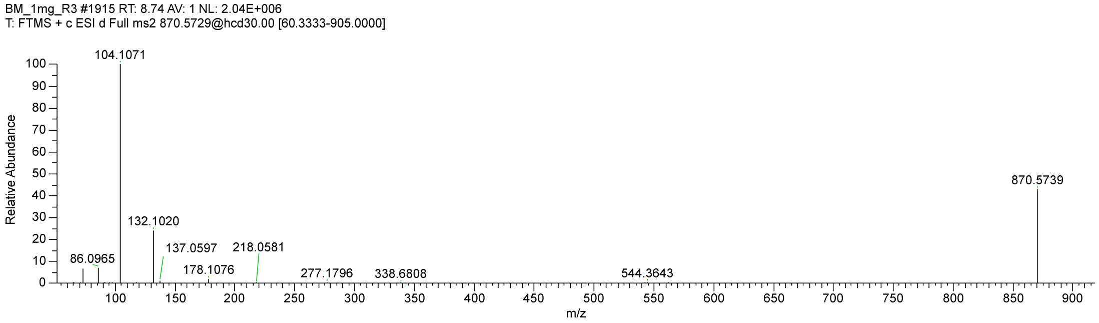

Figure S57. Collision-induced fragmentation spectrum of [**21**+H]$^+$ at *m/z* 870.5731

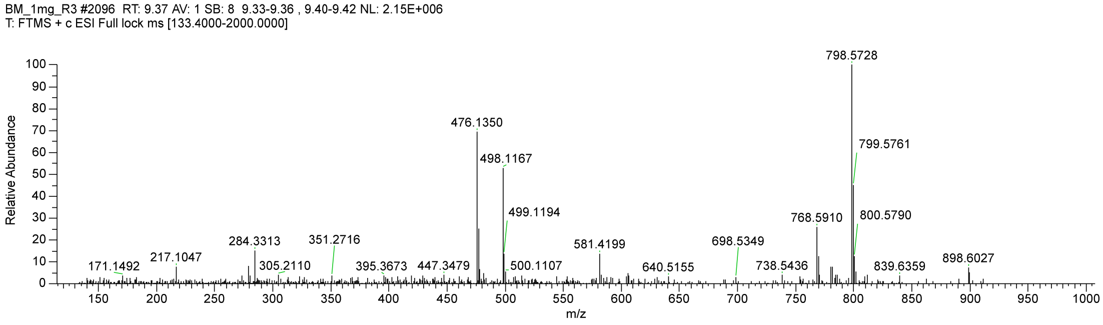

Figure S58. ESI^+-MS spectrum showing ion $[\mathbf{22}+H]^+$ at *m/z* 798.5731

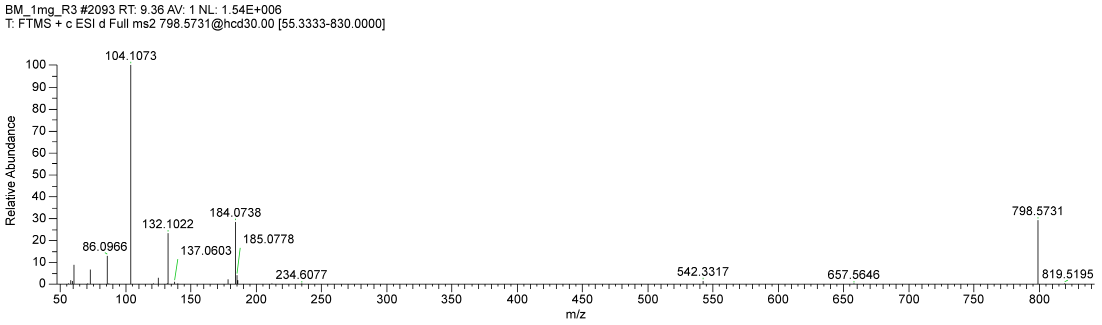

Figure S59. Collision-induced fragmentation spectrum of $[\mathbf{22}+H]^+$ at *m/z* 798.5731

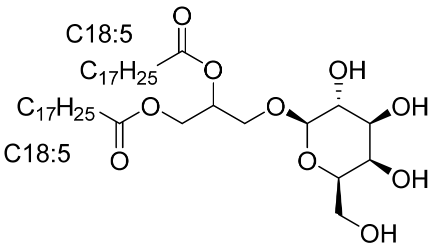

Figure S60. Proposed structure of compound **23**

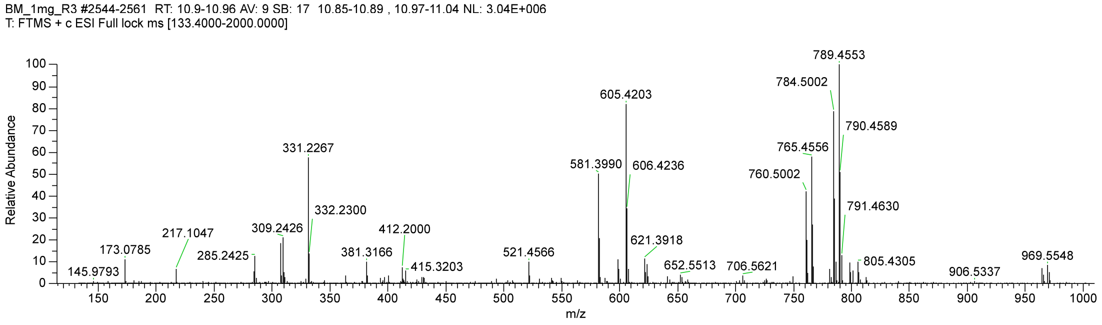

Figure S61. ESI^+-MS spectrum showing ion $[\mathbf{23}+Na]^+$ at *m/z* 789.4553

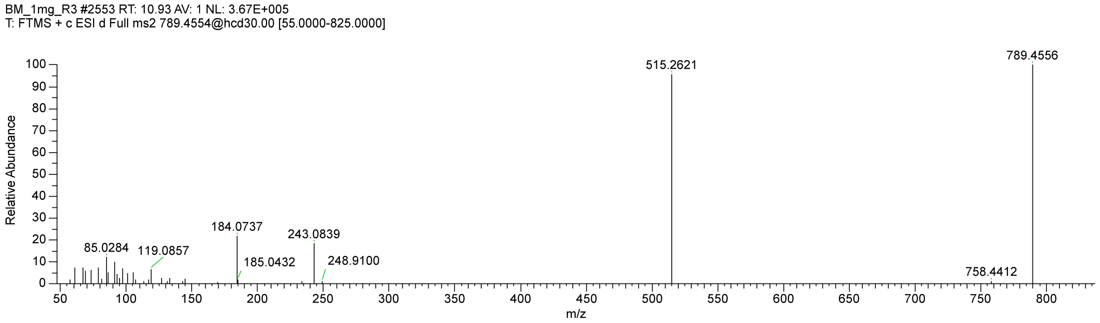

Figure S62. Collision-induced fragmentation spectrum of [**23**+Na]$^+$ at *m/z* 789.4553

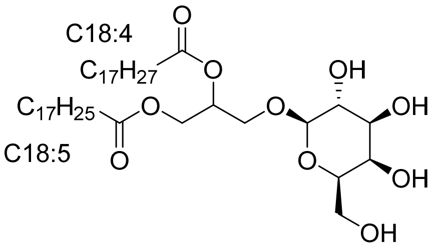

Figure S63. Proposed structure of compound **24**

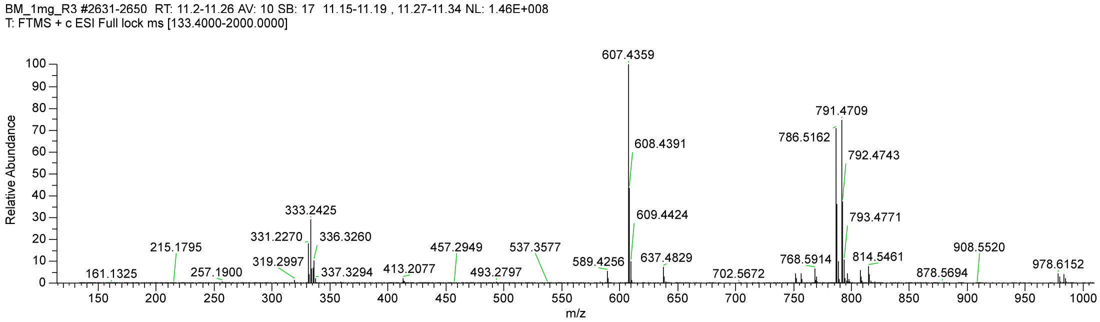

Figure S64. ESI$^+$-MS spectrum showing ion [**24**+Na]$^+$ at *m/z* 791.4709

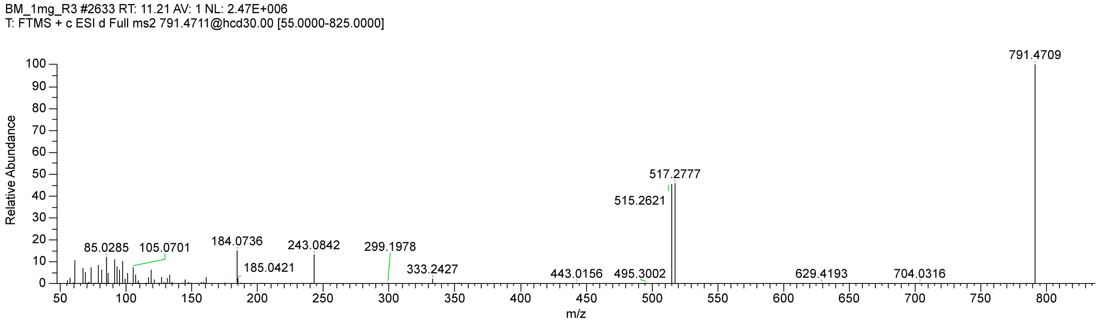

Figure S65. Collision-induced fragmentation spectrum of [**24**+Na]$^+$ at *m/z* 791.4709

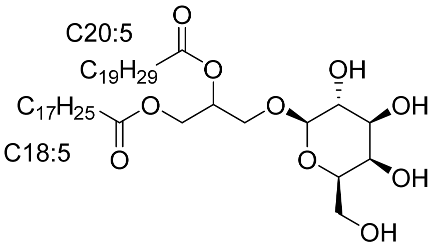

Figure S66. Proposed structure of compound **25**

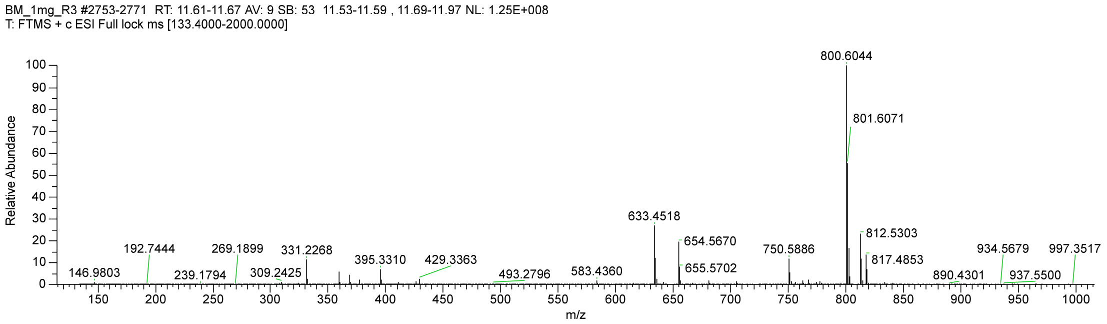

Figure S67. ESI$^+$-MS spectrum showing ion [**25**+Na]$^+$ at *m/z* 817.4853

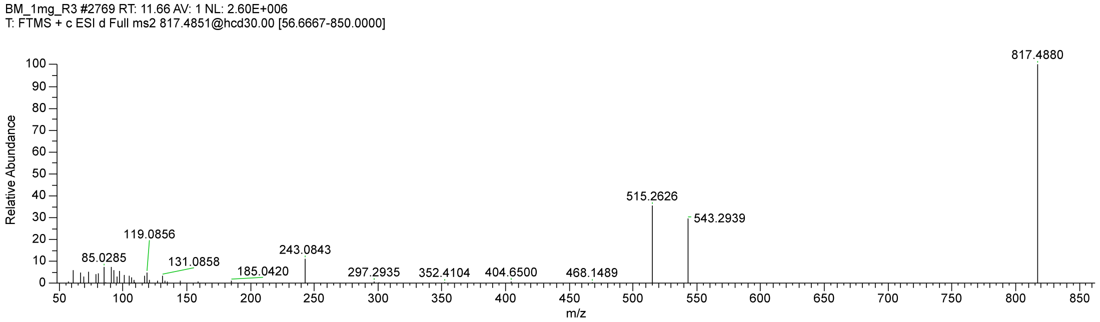

Figure S68. Collision-induced fragmentation spectrum of [**25**+Na]$^+$ at *m/z* 817.4866

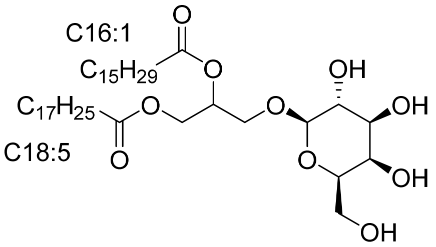

Figure S69. Proposed structure of compound **26**

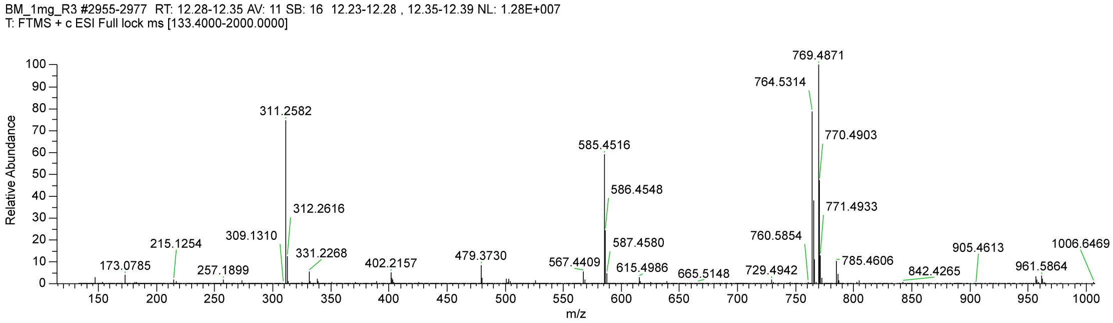

Figure S70. ESI$^+$-MS spectrum showing ion [**26**+Na]$^+$ at *m/z* 769.4871

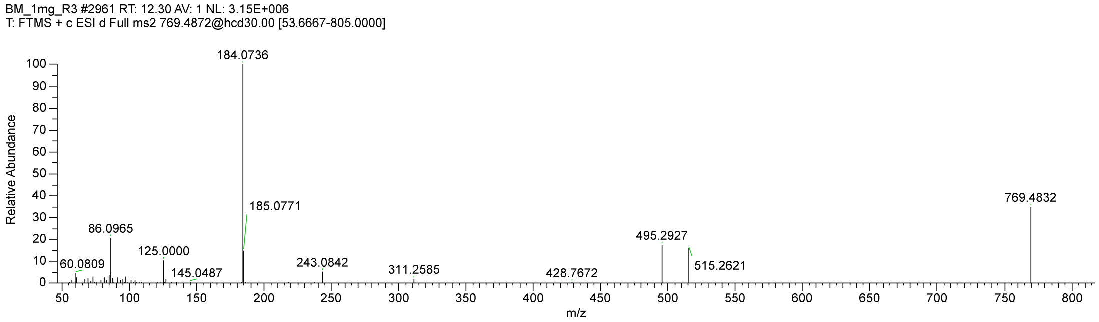

Figure S71. Collision-induced fragmentation spectrum of [**26**+Na]$^+$ at *m/z* 769.4871

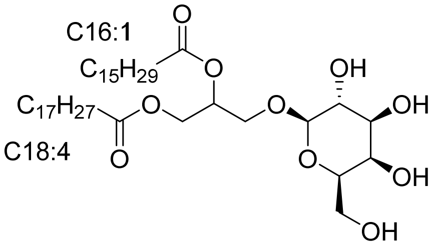

Figure S72. Proposed structure of compound **27**

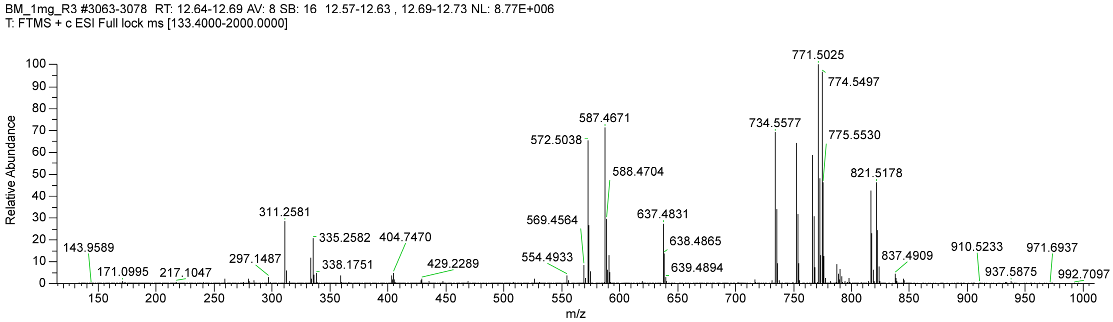

Figure S73. ESI$^+$-MS spectrum showing ion [**27**+Na]$^+$ at *m/z* 771.5025

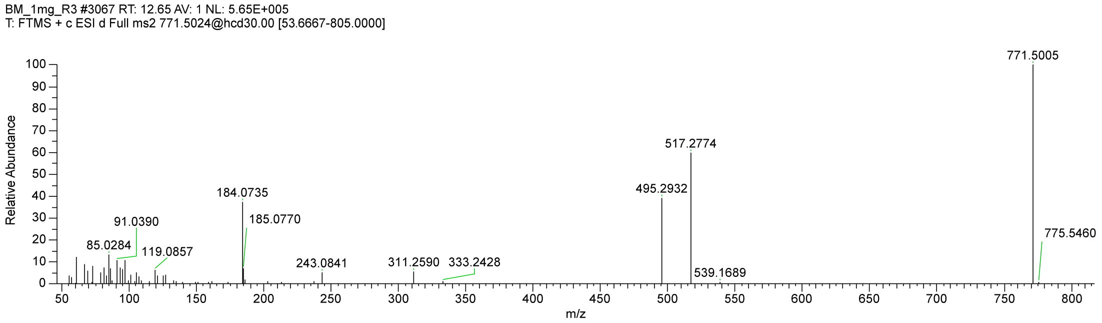

Figure S74. Collision-induced fragmentation spectrum of [**27**+Na]$^+$ at *m/z* 771.5025

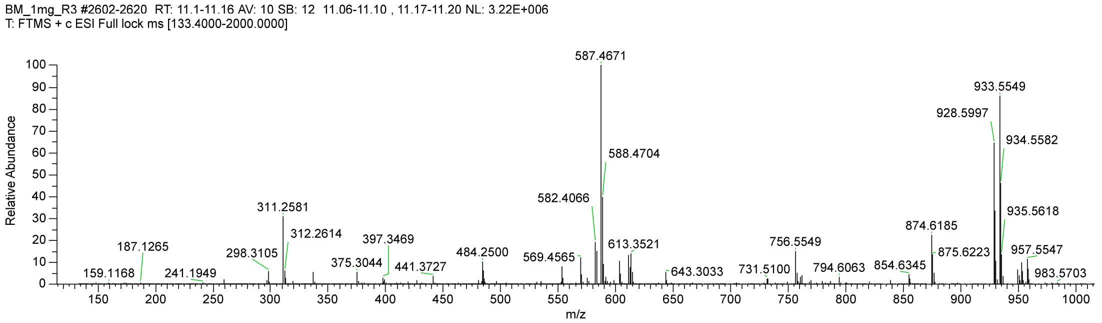

Figure S75. ESI$^+$-MS spectrum showing ion [**28**+H]$^+$ at *m/z* 613.3521

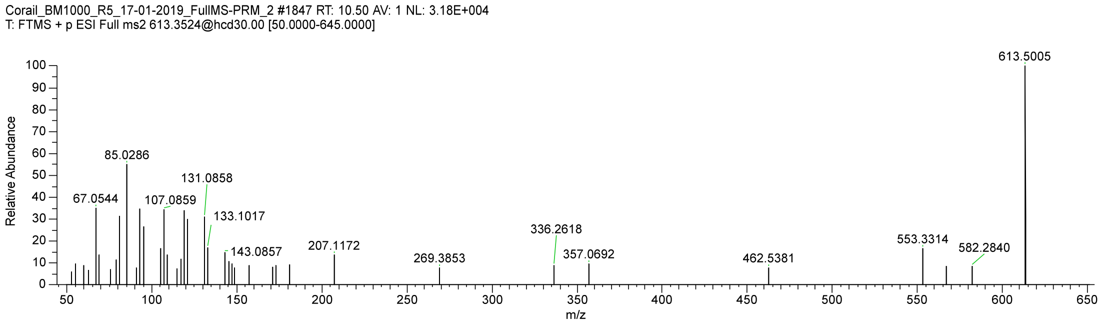

Figure S76. Collision-induced fragmentation spectrum of [**28**+H]$^+$ at *m/z* 613.3521

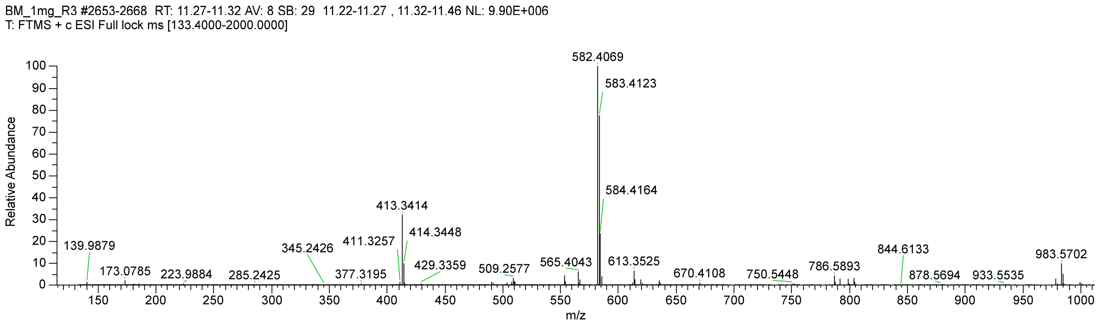

Figure S77. ESI$^+$-MS spectrum showing ion [**29**+H]$^+$ at *m/z* 613.3525

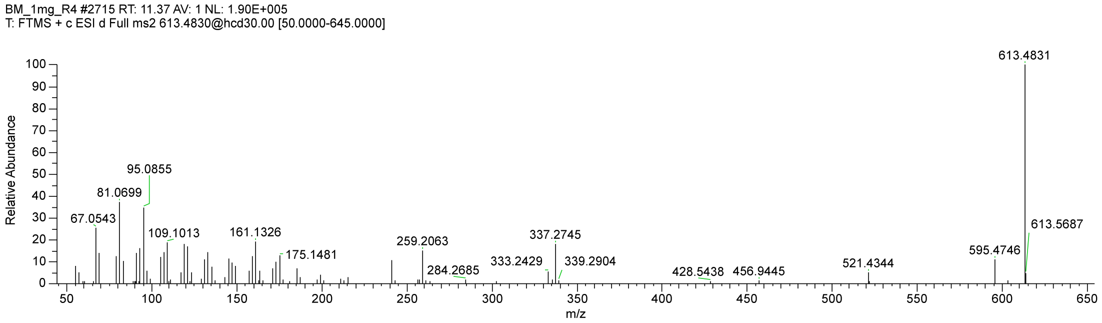

Figure S78. Collision-induced fragmentation spectrum of [**29**+H]$^+$ at *m/z* 613.3521

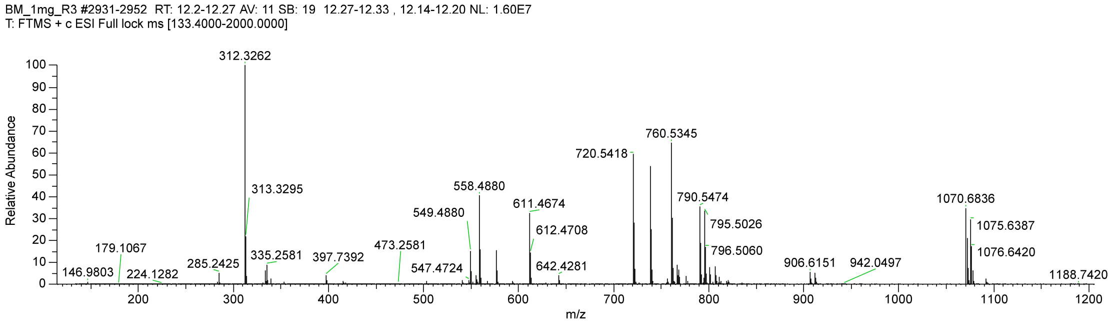

Figure S79. ESI$^+$-MS spectrum showing ion [**30**+NH_4]$^+$ at *m/z* 1070.6836

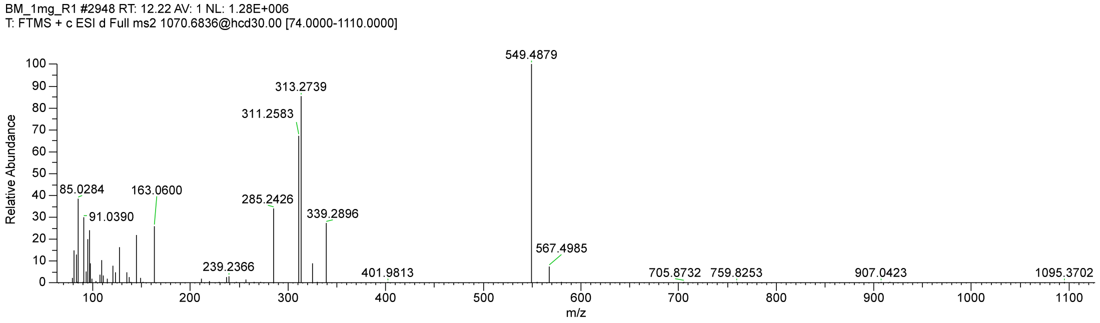

Figure S80. Collision-induced fragmentation spectrum of [**30**+NH_4]$^+$ at *m/z* 1070.6836
